# Electrochemical Investigation of the Stability of Poly-Phosphocholinated Liposomes

**DOI:** 10.3390/molecules29153511

**Published:** 2024-07-26

**Authors:** Miroslav Karabaliev, Boyana Paarvanova, Gergana Savova, Bilyana Tacheva, Sabrina Jahn, Radostina Georgieva

**Affiliations:** 1 Department of Physics, Biophysics, Roentgenology and Radiology, Faculty of Medicine, Trakia University, 11 Armeiska Str., 6000 Strara Zagora, Bulgaria; boyana.parvanova@trakia-uni.bg (B.P.); gergana.savova@trakia-uni.bg (G.S.); bilyana.tacheva@trakia-uni.bg (B.T.); 2 Liposphere Ltd., Aarava 1, Givaat Shemuel 5400804, Israel; sabrina.jahn@lipo-sphere.com; 3 Institute of Transfusion Medicine, Charité-Universitätsmedizin Berlin, Charitéplatz 1, 10117 Berlin, Germany

**Keywords:** poly-phosphocholinated liposomes, hydration lubrication, electrochemical methods

## Abstract

Poly[2-(methacryloyloxy)ethyl phosphorylcholine] liposomes (pMPC liposomes) gained attention during the last few years because of their potential use in treating osteoarthritis. pMPC liposomes that serve as boundary lubricants are intended to restore the natural lubrication properties of articular cartilage. For this purpose, it is important that the liposomes remain intact and do not fuse and spread as a lipid film on the cartilage surface. Here, we investigate the stability of the liposomes and their interaction with two types of solid surfaces, gold and carbon, by using cyclic voltammetry (CV) and electrochemical impedance spectroscopy (EIS). With the aid of a hydrophilic species used as an electroactive probe in the solution, the charge transfer characteristics of the electrode surfaces are obtained. Additionally, from EIS, the capacitance characteristics of the surfaces are derived. No decrease of the peak currents and no displacement of the peak potentials to greater overpotentials are observed in the CV experiments. No decrease in the apparent capacitance and increase in the charge transfer resistance is observed in the EIS experiments. On the contrary, all parameters in both CV and EIS do change in the opposite direction. The obtained results confirm that there is only physical adsorption without fusion and spreading of the pMPC liposomes and without the formation of lipid films on the surfaces of both gold and carbon electrodes.

## 1. Introduction

Osteoarthritis (OA) is a common orthopedic condition from which 7% of the population suffers [1]. It can affect one or more joints and is characterized by the progressive degradation of the articular cartilage that leads to high wear and friction between the articulating joint surfaces. The patient experiences this as pain, which typically limits an active lifestyle. Following the failure of conservative treatment options such as physiotherapy and exercise, local intra-articular (IA) injections are currently the preferred option to treat the disease, as the lack of vasculature within the joints limits systemic treatment success [1,2]. There are two main approaches to treating the disease with IA injections. One is to deliver compounds to reduce pain and inflammation (such as corticosteroids), and the other is to deliver substances that improve joint lubrication and biomechanical functionality. It is assumed that there are two mechanisms by which lubrication can reduce friction between two contacting surfaces. One is hydro-dynamical lubrication by fluid films trapped between the surfaces, and the other is by suitable molecular boundary layers attached to the surfaces. For biological surfaces in aqueous media, such as the articular cartilage surfaces of the joints, lubrication is most probably in the mixed regime, where the two main mechanisms, fluid film and boundary lubrication, act simultaneously [3].

Among the substances that are delivered intra-articularly and claimed to improve the lubrication of the joint are hyaluronic acid (HA) formulations. HA is a natural component of the synovial fluid that plays a major role in its viscoelastic properties. It is approved for use in treating OA by the US Food and Drug Administration and the European Medicines Agency. However, the use of IA HA injections has shown mixed clinical efficacy and some adverse effects, resulting in various governmental bodies not reimbursing HA injections [1].

Phospholipids are another substance that is a natural component of the synovial fluid and, for this reason, are also considered a promising candidate for improving the lubrication in the joint after being delivered by IA injection. Since lipids tend to self-organize in aqueous media, they would form monolayers on the surface, bilayers and multilayers, or micelles. Recently, it has been reported [4] that small unilamellar phosphatidylcholine liposomes self-assemble in close-packed layers on solid surfaces to reduce the coefficient μ of sliding friction between them μ ≈ 1 × 10^−4^–2 × 10^−5^, at pressures up to at least ca. 12 MPa [4]. The hydration lubrication mechanism discovered by J. Klein [5,6,7] is proposed to rule the frictional performance of the liposomes on surfaces at pressure conditions similar to those present in the major joints. Furthermore, liposomes were modified with lipid–polymer conjugates, which create “hydration hotspots” in order to increase the liposomal, structural stability, and retention time in joints [8,9].

These liposomes are referred to as poly-phosphocholinated liposomes (pMPC liposomes). They contain lipid–polymer conjugates (LPC) where the tail of the lipid is attached to a poly[2-(methacryloyloxy)ethyl phosporylcholine] (pMPC) polymer carrying phosphocholine-like monomers on its backbone [9]. pMPC was synthesized as a novel blood-compatible polymer and was known to suppress reactions such as protein adsorption and cell adhesion due to its highly hydrated nature [10]. The MPC repeating units have a unique structure composed of zwitterionic monomers, which resemble the natural lipid headgroups and are, therefore, considered to be “bio-inspired” [1]. A recently published study discovered that pMPCylated liposomes are biodegraded by M2 macrophages in the joint following local injection, as confirmed using a triple-label immunofluorescent (IF) assay [11].

To be effective as a boundary lubricant on a biological surface for a prolonged period of time, the liposome is designed to remain intact without spreading on the surface to form a lipid mono- or bilayer film. The present study is aimed at investigating the structural stability of pMPC liposomes on solid surfaces.

Electrochemical methods deal with processes that happen on the surface of electrodes immersed in electrolyte solutions. The electrochemical cell under investigation usually comprises three electrodes: a working electrode, a reference electrode, and an auxiliary electrode. The type, size, and position of each of the three electrodes are such that the electric current flowing through the cell depends on the electric potential and the processes on the surface of the working electrode. The processes are an accumulation of charges on the surface and charge transfer through the surface. Both processes depend on the properties of the bulk electrolyte, such as the concentration of ions and concentrations and diffusion coefficients of some electroactive species, as well as the properties and conditions of the electrode surface. In this respect, electrochemical methods appear promising for the investigation of eventual physical or chemical adsorption, fusion, and spreading of liposomes on the electrode surface, which would significantly affect the two processes mentioned above [12].

For the purpose of this study, we used two electrochemical methods: cyclic voltammetry (CV) and electrochemical impedance spectroscopy (EIS). Both methods are widely used in the investigation of lipid films spreading on the surface of electrodes [13,14,15,16,17,18,19].

CV is an electric potential sweep method in which the potential is controlled, and the resulting current is measured [12]. In CV, the potential is changed linearly in positive and then in negative directions around the standard potential (*E*^0^) of an electroactive couple present in the solution. The electroactive couple comprises the oxidized (O) and the reduced (R) form of a species that turn into each other when electrons are exchanged with the electrode surface. The shape of the curve in the voltammogram depends on the heterogeneous rate constants for reduction and oxidation (*k*_f_ and *k*_b_) and the rate of the mass transfer of the species from the bulk to the electrode surface due to diffusion. If the charge transfer reaction is fast, the reaction is limited by the mass transfer, and it is called a diffusion-limited reaction. With diffusion-limited reactions, the oxidation and reduction current peaks are observed at potentials that are close to the equilibrium potential of the redox reaction (*E*^0′^). On the other hand, if the electron transfer through the electrode surface is slow (small values of *k*_f_ and *k*_b_) compared to the rate of diffusion of the species from the bulk to the electrode surface, the overall process is limited by the electron transfer. In this case, the process is kinetically limited. Correspondingly, the oxidation and reduction peaks are shifted to greater overpotentials—the oxidation peak is shifted to a potential more positive than the equilibrium potential, and the reduction peak is shifted to a more negative potential than the equilibrium potential. The difference between the oxidation peak potential and the reduction peak potential increases, and the peak currents become smaller [12,20].

Because the shape of the voltammograms strongly depends on the conditions at the electrode surface, CV is one of the preferred methods to study the formation and properties of lipid films on surfaces.

A common method of preparation of lipid films on solid surfaces is based on the fusion and spreading of liposomes [13,21]. The method consists of incubation of the solid surface object, e.g., electrode, in a suspension of liposomes. Depending on the type of the electrode surface, the lipid composition of the liposomes, and the content of the electrolyte solution, the liposomes can adsorb on the surface and eventually start fusing and spreading, eventually forming a lipid film [21,22,23].

The intactness of the lipid films and the presence or absence of bare uncovered lipids regions on the electrode surface is tested using CV, adding a small quantity of hydrophilic electroactive species to the main supporting electrolyte (usually ferri/ferrocyanide Fe(CN)_6_^3−^/Fe(CN)_6_^4−^ in millimolar concentration) [18,19,24]. Depending on the intactness of the lipid film, the obtained voltammogram can fit one of the following (compared with the voltammogram obtained on bare electrode):

(1) Voltammogram that is the same as that obtained on the bare electrode. This is when the liposomes do not form any kind of lipid layer on the surface, do not hamper the electron transfer between the hydrophilic electroactive species and the electrode, as well as do not change the effective area for electron transfer of the electrode.
(2) Similar voltammograms as those obtained on bare electrodes will also be obtained when the liposomes are physically adsorbed on the electrode surface but do not fuse and spread and do not form lipid layer regions on the electrode surface. In this case, the effective area of the electrode will not be altered or altered to a small extent, bearing in mind the spherical shape of the liposomes and the small contact area between them and the surface.
(3) Voltammogram with oxidation and reduction peaks that are at the same potentials but the peak currents are smaller [18,24]. This happens if the electrodes are partially covered with a lipid film or if a lipid film with defects (or a kind of pores) is formed [18]. In this case, the hydrophilic electroactive species reach the electrode surface in the uncovered regions (or through the pores). Because the electron transfer is not hampered in these areas, the peak potentials are not displaced, and the voltammogram has the typical shape of those of diffusion-limited reactions. However, because the effective area of the electrode is now smaller, the peak currents are smaller.
(4) Voltammogram with peaks that are smaller and displaced to greater peak overpotentials [19]. This happens when the electrode is covered with intact lipid film with no defects at all or with pinhole defects [25]. In this case, the hydrophilic electroactive species cannot reach the electrode surface, and the electron transfer, if any, can happen only via the tunneling effect through the lipid phase. This leads to a significant decrease in the rate constants, and the voltammogram has the typical shape obtained from kinetically limited reactions. This situation can occur with either films with lipids physically adsorbed on the surface [19] or films with lipids chemically adsorbed on the surface [25].

The basic idea of electrochemical impedance spectroscopy (EIS) is to measure the electrical impedance of the electrochemical cell at various frequencies of the alternating electric potential. The experimental impedance data are fitted with an equivalent electrical model comprising several electrical elements such as resistances and capacitances. Each of these elements stands for some of the physical processes in the electrochemical cell. The reference electrode is ideally nonpolarizable, and the auxiliary counter electrode has a larger surface area. Therefore, their impedance is small, and the whole impedance is attributed to the processes at the working electrode and in the electrolyte solution, which is in contact with it [12].

One of the main benefits of EIS is that along with charge transfer characteristics (represented by a charge transfer resistance, *R*_ct_), the capacitive behavior of the working electrode can also be quantified. Being expressed by the capacitance of the double layer *C*_d_, it is strongly affected by any kind of deposits on the electrode surface. The presence of a lipid phase on the surface strongly decreases the apparent capacitance of *C*_d_ or *C*_CPE_. The capacitance of clean bare solid electrodes is usually in the range of 10 to 40 μF/cm^2^, while the apparent capacitance of an electrode covered with lipid film is in the range of 1–2 μF/cm^2^ [26,27,28]. Even in the case of a partially covered gold electrode, the capacitance decreases after liposome spreading from 30 μF/cm^2^ to about 7 μF/cm^2^, which is still a significant decrease [29].

In this study, we investigated the interaction of pMPC liposomes with two different types of solid surfaces, carbon and gold. The two types of surface were chosen because of their different hydrophilicity, the gold being more hydrophilic [30,31]. We used CV and EIS to determine whether the liposomes remain intact on the surface or fuse and spread to form a lipid monolayer or bilayer. The results obtained with pMPC liposomes were compared with results obtained with conventional phosphatidylcholine liposomes (PC liposomes).

## 2. Results and Discussion

The hydrodynamic size of the pMPC liposomal formulations was calculated by the cumulant analysis of the autocorrelation curves and yielded a mean hydrodynamic diameter of 160 to 170 nm and a polydispersity index of around 0.09, which corresponds to a single-size population with a narrow size distribution. For the control PC, liposome size and polydispersity indexes were in the same range. The measurement of the zeta potential in PBS delivered values between −1.5 and −2.8 mV for the pMPC liposomes and −5.8 to −8.0 mV for the control PC liposomes. These values are characteristic of electrically neutral particles and of slightly negatively charged particles, respectively.

The pMPC liposomes were also characterized concerning their lamellar structure using a fluorescence quenching assay [32] as described in Supplementary Materials. The results confirmed a highly unilamellar formulation with 43% quenching of the fluorescence before the addition of a crosslinker to achieve permeabilization of the liposomes (Figures S1 and S2).

All electrochemical experiments were performed with parallel measurements with two types of solutions: a solution without liposomes and another solution containing liposomes. The supporting electrolyte for both was PBS (Dulbecco’s phosphate buffered saline without calcium and magnesium, pH 7.4). The ferri/ferrocyanide couple Fe(CN)_6_^3−^/Fe(CN)_6_^4−^ was added to each solution to test the electron transfer through the electrode surface.

The investigations were carried out with two types of screen-printed electrodes, gold electrodes, and carbon electrodes, and in two series.

In the first series, a drop of the experimental solution (either with or without pMPC liposomes) was placed on the screen-printed electrodes, and the impedance was measured every two minutes over 20 min. At the 22nd minute, a CV was measured. In the case of solutions with liposomes, the spreading of liposomes on the electrode surface would result in changes in the electrode characteristics and should be expected within the first few minutes of incubation [29,33,34].

In the second series, electrodes were incubated for 24 h in a solution containing pMPC liposomes. Other electrodes were incubated in solutions without liposomes for comparison. After 24 h, the impedances and CV were measured and compared. In the 24-h experiments, third parallel measurements were taken by incubating the electrodes in a solution containing liposomes prepared with conventional unmodified phosphatidylcholine (PC).

### 2.1. Gold Electrodes

**Figure 1:**
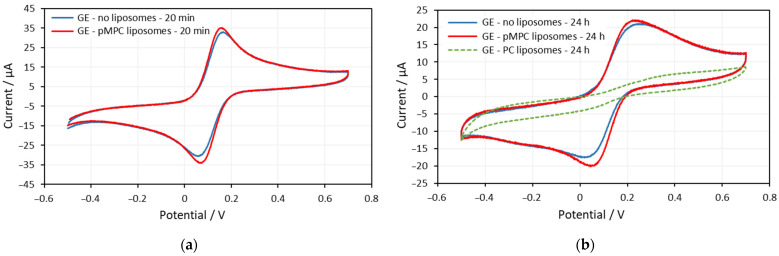
(**a**) Cyclic voltammograms on gold electrodes (GE) at the 22nd minute of solution of equimolar mixture of 0.5 mM ferricyanide (Fe(CN)_6_^3−^) and 0.5 mM ferrocyanide (Fe(CN)_6_^4−^) in the absence (blue curve) and presence of pMPC liposomes (red curve). Screen-printed gold electrodes: DRP-220AT. Supporting electrolyte: PBS. Scan rate: 0.2 V/s. (**b**) Cyclic voltammograms on gold electrodes at the 24th hour of equimolar solution of 0.5 mM ferricyanide (Fe(CN)_6_^3−^) and 0.5 mM ferrocyanide (Fe(CN)_6_^4−^) in the absence (blue curve) and presence of pMPC liposomes (red curve) and of PC liposomes (green curve). Screen-printed gold electrodes: DRP-220AT. Supporting electrolyte: PBS. Scan rate: 0.2 V/s.

Figure 1a shows voltammograms on screen-printed gold electrodes obtained at the 22nd minute in solutions without liposomes and with pMPC liposomes. The CVs on gold electrodes obtained after 24 h of incubation in PBS, in pMPC solution, and in PC solution are shown in Figure 1b.

The voltammogram on the gold electrode obtained in solution with pMPC liposomes overlaps with the voltammogram obtained in solution without liposomes (Figure 1a). The potentials of the oxidation and reduction peaks are the same, as well as the height of the peaks’ currents. There is a very small difference in the peaks’ currents, but these are the peaks with pMPC liposomes that are slightly larger. All this suggests that the liposomes do not form any kind of lipid layer on the gold surface, do not hamper the electron transfer, as well as do not change the effective area for electron transfer of the electrode.

After 24 h of incubation, the voltammograms in the solution without liposomes and the solution with pMPC liposomes are also almost the same (Figure 1b). Moreover, the voltammogram of the pMPC liposome solution has slightly greater peak currents (the red curve) than the one obtained in solution without liposomes (the blue curve). This means that the liposomes did not spread on the surface and did not form any lipid film on the electrode surface after 24 h of incubation. Completely different is the voltammogram obtained after 24 h of incubation in solution with PC liposomes (the green curve in Figure 1b). Both the oxidation and reduction peaks almost disappeared but are still noticeable at the same electrode potentials. This is the third situation described in the Introduction section. As a result of adsorption, fusion, and spreading of the PC liposomes on the gold surface of the electrode, a lipid film with some small defects is formed.

**Figure 2:**
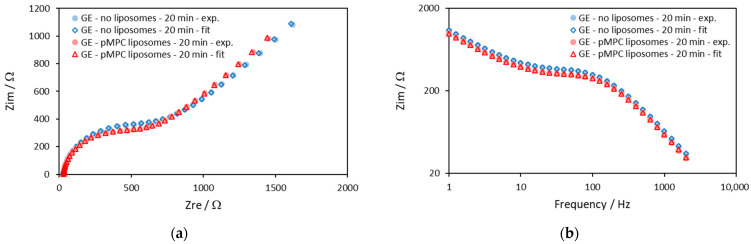
Impedance diagrams on gold electrodes of solution of an equimolar mixture of 0.5 mM ferricyanide (Fe(CN)_6_^3−^) and 0.5 mM ferrocyanide (Fe(CN)_6_^4−^) in the absence (blue symbols) and in the presence of PMPC liposomes (red symbols) after 20 min of incubation with the solutions. The solid symbols are the experimental data, and the open symbols are the results from the fitting procedure with the model shown in Figure 3. Screen-printed gold electrodes: DRP-220AT; surface area, 0.1257 cm^2^. Supporting electrolyte: PBS. Frequency range: 1–2000 Hz. DC potential: 95 mV. (**a**) Nyquist plot—*Z*_im_ vs. *Z*_re_; (**b**) Bode plot—*Z*_im_ vs. Frequency.

Figure 2 shows impedance diagrams that are obtained with gold electrodes after 20 min of incubation in a solution without liposomes and another in a solution with pMPC liposomes. The impedances were measured at the DC potential of the working electrode against the reference electrode 95 mV, which was the apparent equilibrium potential of the redox reaction measured from the voltammograms in Figure 1.

The two typical regions of the Randles circuit model are well seen in the Nyquist plot (Figure 2a)—the semicircle region and the Warburg line region. However, it is important to note that the impedance curves obtained with pMPC liposomes are identical to those obtained without liposomes, suggesting a lack of interaction between the pMPC liposomes and the gold surface.

**Figure 3:**
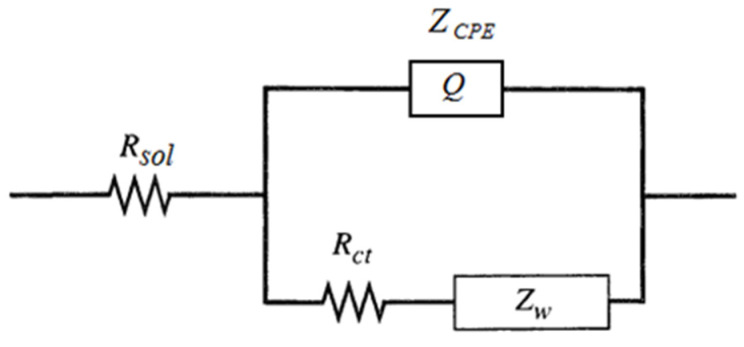
Equivalent circuit with *Z*_CPE_ in parallel with *R*_ct_ and *Z_w_* used in the work to fit the experimental data.

All impedance data on gold electrodes are fitted with the model that is shown in Figure 3.

The electrical model is a modified Randles circuit in which the electrical double-layer capacitance is replaced with a constant phase-angle element (CPE). The modified Randles model comprises the resistance of the electrolyte solution *R*_sol_ in series with the working electrode impedance that combines three elements, a constant phase-angle element (denoted with *Q*), a charge transfer resistance *R*_ct_ and a Warburg impedance *Z_w_*. The model is described in details in Section 3.

In Figure 2, the solid symbols are the experimental data, and the open symbols are the results of the fitting procedure. Figure 2 shows that the fitting procedure with the model gives a very good approximation.

The parameters that are calculated by the fitting procedure for the data shown in Figure 2 are shown in Table 1.

The data in Table 1 suggest that there is a very small rather negligible effect of the liposomes on the impedance parameters of gold electrodes. The observed differences of *Q* and *R*_ct_ taken in combination cannot be related to liposome spreading and fusion and formation of lipid film on the surface nor to deposits of lipid molecules on the electrode surface. Formation of regions with lipid film on the surface would increase *R*_ct_ and decrease *Q* at the same time, which is not observed in our experiments.

**Figure 4:**
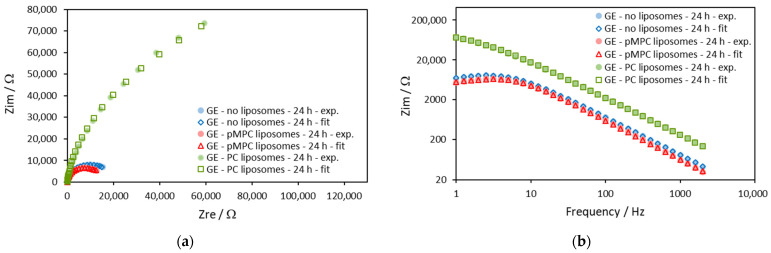
Impedance diagrams on gold electrodes at the 24th hour of equimolar solution of 0.5 mM ferricyanide (Fe(CN)_6_^3−^) and 0.5 mM ferrocyanide (Fe(CN)_6_^4−^) in the absence (blue symbols) and in the presence of pMPC liposomes (red symbols) and of PC liposomes (green symbols). The solid symbols are the experimental data, and the open symbols are the results from the fitting procedure with the model shown in Figure 3. Screen-printed gold electrodes: DRP-220AT. Supporting electrolyte: PBS. Frequency range: 1–2000 Hz. (**a**) Nyquist plot—*Z*_im_ vs. *Z*_re_; (**b**) Bode plot—*Z*_im_ vs. Frequency.

The impedances on gold electrodes obtained after 24 h of incubation are shown in Figure 4.

Comparing the semicircles in Figure 4a, we can see that the semicircle obtained with pMPC liposomes in solution has a similar but slightly smaller diameter compared with the semicircle of the impedance obtained in solution without liposomes (the blue and the red markers). This means slightly smaller charge transfer resistance, *R*_ct_, when the gold electrode is incubated with pMPC liposomes. In the higher frequencies of the Bode plot (Figure 4b), the curve of the electrode with the pMPC liposomes is beneath the one without liposomes, meaning higher apparent capacitance with pMPC liposomes. On the other hand, the data for the impedance of gold electrodes incubated 24 h in solution with PC liposomes are very different (the green markers). On the Nyquist plot (Figure 4a), the data look like a section of semicircle but with a much bigger diameter compared to the semicircles of the impedances without liposomes and with pMPC liposomes. This suggests a bigger charge transfer resistance, *R*_ct_, when the gold electrode is incubated with PC liposomes. In the Bode plot (Figure 4b), the curve of the electrode with the PC liposomes is much above the two other curves, meaning quite a smaller apparent capacitance after incubation with PC liposomes.

The parameters of the impedances obtained by the approximation with the model are given in Table 2.

**Figure 5:**
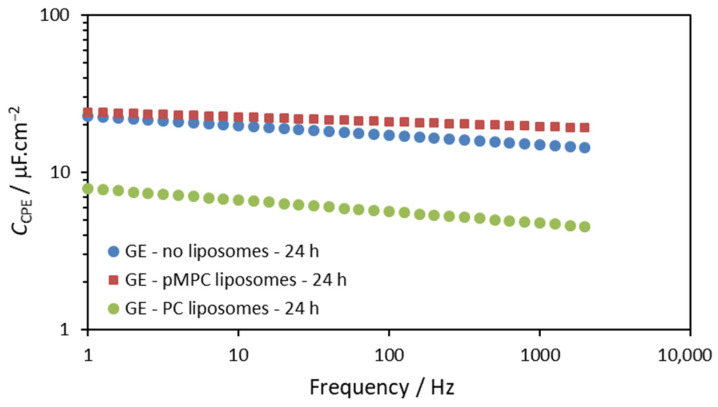
Apparent capacitance of the gold electrode (GE) after 24 h of incubation calculated from the fitting parameters *Q* and *α*. Blue circles—in the absence of liposomes; red squares—in the presence of pMPC liposomes; green circles—in the presence of PC liposomes. Measurements condition as in the Figure 4 caption.

After 24 h of incubation, the *Q* parameter slightly decreased for the electrodes in solutions without liposomes and with pMPC liposomes. It is noted that the values of *Q* after 24 h are very close for the two solutions—with pMPC liposomes and without liposomes. This suggests that the observed changes in the capacitive behavior (represented by the parameter *Q*) are due to some adsorption of ions from the PBS on the gold surface rather than to the interaction of the pMPC liposomes with the gold surface. Most probably, these are the phosphate ions in the PBS solution [35]. The result for the *Q* parameter of the gold electrode incubated in solution with PC liposomes shows that it is much smaller, suggesting a different type of interaction of the PC liposomes compared with the pMPC liposomes. In order to represent these results more clearly, we calculated the apparent capacitance of the gold electrodes for the three cases using Equation (2) and obtained values of the *Q* and α parameters. The results for such calculation are presented in Figure 5.

The *Q* parameter is very similar for the cases without liposomes and with pMPC liposomes, but the apparent capacitance, calculated by Equation (2) from the *Q* and α parameters, is bigger for the solution with pMPC liposomes for all frequencies used in the measurements. 

As can be seen in Figure 5, after 24 h of incubation, the presence of pMPC liposomes results in slightly bigger but similar apparent capacitance for all frequencies used in the measurements compared to incubation in pure PBS. This suggests that no spreading and further interactions of pMPC liposomes occurred on the gold surface. On the other hand, the apparent capacitance of the electrode after 24-h incubation in solution with PC liposomes is remarkably smaller, indicating the formation of lipid film on the surface in this case. The apparent capacitance of this PC-modified gold electrode is in the range of 4.5–7.9 μF/cm^2^. This value is bigger than a value of 1–2 μF/cm^2^ that is expected for intact defect-free lipid mono- or bilayer spread on the electrode surface [26,27,28]. Obviously, a lipid film with some defects is formed after the incubation with PC liposomes [29]. This is also confirmed by the results of the CV presented and commented on above in the text (Figure 1b)

Analyzing the values of the charge transfer resistance, *R*_ct_, several points are worth mentioning.

First, *R*_ct_ increases after 24 h of incubation in all solutions, without liposomes and with pMPC or PC liposomes. An increase of 100-fold to 140-fold of *R*_ct_ is observed in pMPC liposome solution and in PBS, respectively. In PC liposome solution, an 800-fold increase of *R*_ct_ is noticed compared with *R*_ct_ in PBS, which is measured immediately after the contact of the buffer and the electrode (0 min of incubation).

Second, the *R*_ct_ with pMPC liposomes is smaller but close to the *R*_ct_ in PBS when in both solutions, the incubation is 24 h. In both cases, with and without pMPC liposomes, an almost equivalent increase of *R*_ct_ is observed. This could be attributed to the adsorption of ions from the PBS [35] and not to the spreading of pMPC liposomes and the formation of lipid film on the electrode surface, as in the latter case, the opposite differences in the parameters would be observed. Changes in capacitance, as well as an increase of the charge transfer resistance due to the interaction of the gold surface with PBS, were reported by other authors after 30 min of incubation of the gold electrodes in PBS [34]. The slightly smaller *R*_ct_ with pMPC liposomes corresponds to the slightly bigger peak current in CV (Figure 1b). Both the smaller *R*_ct_ and bigger peak current in CV could be explained by an increased concentration of the electroactive species Fe(CN)_6_^3−/4−^ near the electrode surface in the presence of pMPC liposomes. The observed increase of the Warburg parameter W could also be explained by increased concentration of the electroactive species Fe(CN)_6_^3−/4−^ near the electrode surface (Equations (6) and (8)). Such increased concentration should be attributed to the interaction of Fe(CN)_6_^3−/4−^ with the highly hydrated surface of the pMPC liposomes. As mentioned in the Introduction section, the MPC repeating units have a unique structure composed of zwitterionic monomers, which increases the polarity and creates a highly hydrated layer near the pMPC liposome surface. It could be expected that multivalent ions such as Fe(CN)_6_^3−/4−^ would interact with this hydrated polar layer, somewhat increasing their concentration in the vicinity of the pMPC liposomes. Physical adsorption of the pMPC liposomes on the gold surface would increase the Fe(CN)_6_^3−/4−^ concentration near the electrode surface. On the other hand, chemical adsorption of the pMPC liposomes should be excluded as, in such case, the chemical bonds would decrease the sites on the electrode surface available for the electron charge transfer, and the result would be decreased charge transfer and greater charge transfer resistance *R_ct_*.

Third, as expected in the case of lipid film formation on the electrode surface, the charge transfer resistance *R*_ct_ is significantly bigger in the PC liposome solution. It is 5.9 times bigger compared to the electrode incubated in PBS and 7.6 times bigger compared to the electrode incubated in pMPC liposome solution. This is in corroboration with all other parameters obtained with CV and EIS for gold electrodes in PC liposome solution. It is obvious that the PC liposomes form a lipid film on the gold surface in contrast with the pMPC liposomes that remain stable and intact and do not spread on the gold surface.

### 2.2. Carbon Electrodes

**Figure 6:**
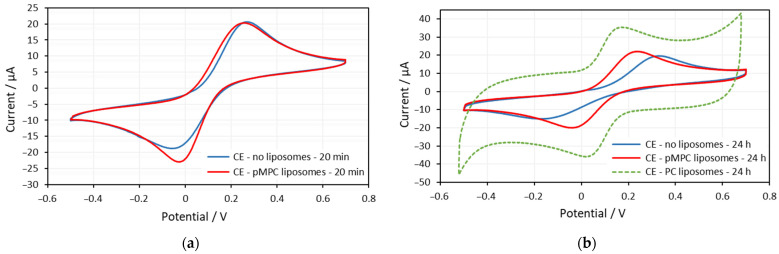
(**a**) Cyclic voltammograms on carbon electrodes (CE) at the 22nd minute of equimolar solution of 0.5 mM ferricyanide (Fe(CN)_6_^3−^) and 0.5 mM ferrocyanide (Fe(CN)_6_^4−^) in the absence (blue curve) and presence of pMPC liposomes (red curve). Screen-printed carbon electrodes: DRP-110. Supporting electrolyte: PBS. Scan rate: 0.2 V/s. (**b**) Cyclic voltammograms on carbon electrodes at the 24th hour of equimolar solution of 0.5 mM ferricyanide (Fe(CN)_6_^3−^) and 0.5 mM ferrocyanide (Fe(CN)_6_^4−^) in the absence (blue curve) and presence of pMPC liposomes (red curve) and of PC liposomes (green curve). Screen-printed carbon electrodes: DRP-110. Supporting electrolyte: PBS. Scan rate: 0.2 V/s.

Figure 6a presents the voltammograms on screen-printed carbon electrodes obtained at the 22nd minute with solutions without liposomes and with pMPC liposomes. The CVs on carbon electrodes obtained after 24 h of incubation are shown in Figure 6b. Along with voltammograms obtained with carbon electrodes incubated in PBS without liposomes and in solution with pMPC liposomes in Figure 6b are shown results of CV obtained with carbon electrode incubated 24 h in a solution containing PC liposomes.

The two voltammograms in Figure 6a overlap to a great extent. The potentials of the oxidation and reduction peaks are the same. The oxidation peak currents are the same as well. The difference in reduction peak currents is small, and even the peak with pMPC liposomes is slightly larger. All this suggests that this is the first or the second situation previously described in the Introduction section above when the voltammogram with liposomes is the same as that obtained on the bare electrode. This is when the liposomes do not form any kind of lipid layer on the surface, do not hamper the electron transfer, and do not change the effective area for electron transfer of the electrode. From this result only, it cannot be concluded whether the liposomes adsorb physically on the surface or not.

Figure 6b presents CV obtained on gold electrodes after 24 h incubation in PBS (blue curve), solution with pMPC liposomes (red curve), and solution with PC liposomes. It can be seen that the difference between the three voltammograms is quite noticeable.

Comparing the CV in PBS and in pMPC liposome solution, the data show that the electron transfer is more prominent with pMPC liposomes in solution. On the CV diagram in Figure 6b, the voltammogram obtained with pMPC liposomes has higher peak currents, and the difference between the peak potentials is smaller than in the voltammogram obtained in PBS without liposomes. This suggests faster electron transfer in solutions with pMPC liposomes compared to the solution without liposomes. It should also be noted that the peak positions and heights for pMPC liposomes are almost the same after 20 min of incubation and after 24 h of incubation (the red curves in Figure 6a,b).

The CV curve of the carbon electrode incubated in solution with PC liposomes exhibits bigger oxidation and reduction peaks compared to the two other CVs (Figure 6b). However, it could be noticed that this is due to very large background currents, and the peak heights measured from the backgrounds are similar to the peak heights in the pMPC liposomes voltammogram. As it is known, the background currents in CV are capacitive currents that arise from the charging of the electrode surface without transferring electric charges through it. This suggests a strong interaction of the PC liposomes with the carbon electrode surface that results in an increase of the electrode capacitance without hampering the electron charge transfer.

**Figure 7:**
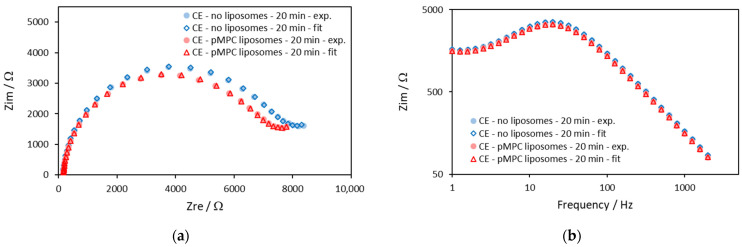
Impedance diagrams on carbon electrodes of equimolar solution of 0.5 mM ferricyanide (Fe(CN)_6_^3−^) and 0.5 mM ferrocyanide (Fe(CN)_6_^4−^) in the absence (blue symbols) and in the presence of pMPC liposomes (red symbols) after 20 min of incubation with the solutions. The solid symbols are the experimental data, and the open symbols are the results from the fitting procedure with the model shown in Figure 3. Screen-printed carbon electrodes: DRP-110; surface area, 0.1257 cm^2^. Supporting electrolyte: PBS. Frequency range: 1–2000 Hz. DC potential: 95 mV. (**a**) Nyquist plot—Z_im_ vs. Z_re_; (**b**) Bode plot—Z_im_ vs. Frequency.

In Figure 7, impedance diagrams are shown, which are obtained after 20 min of incubation in a solution without liposomes and another in a solution with pMPC liposomes. The impedances were measured at DC potential of the working electrode against the reference electrode 95 mV that was the apparent equilibrium potential of the redox reaction measured from the voltammograms in Figure 6.

The two typical regions of the Randles circuit model are seen in the Nyquist plot (Figure 7a)—the semicircle region and the Warburg line region. The straight line of the Warburg region is much less prominent compared to the impedance diagrams obtained on the gold electrodes. The reason for this is the slower electron transfer through the carbon surface and the bigger semicircle with diameter *R*_ct_.

The diameters of the semicircles are almost the same. The diameter of the impedance curve obtained with pMPC liposomes is slightly smaller, which means that the presence of pMPC liposomes does not increase the charge transfer resistance. The curves in the Bode plot (Figure 7b) also largely overlap, especially in the higher frequencies’ region. Since this region is related mostly to the capacitance of the electrode, this suggests that the capacitance is not affected by the presence of liposomes, and there is no spreading of liposomes on the electrode surface or any kind of chemical interaction.

All impedance data are fitted with the same model shown in Figure 3. Again, it is noted that the fitting procedure with the model gives a very good approximation.

The parameters that are calculated by the fitting procedure for the data shown in Figure 7a are presented in Table 3.

The data in Table 3 suggest that there is a very small effect of the pMPC liposomes on the impedance parameters. These effects cannot be related to any kind of pMPC liposome spreading and fusion and formation of lipid film on the surface nor to deposits of lipid molecules on the electrode surface. The parameters *Q* and *α* are almost identical, with a slight increase of *Q* with pMPC liposomes, which means that the capacitive behavior is not affected by the pMPC liposomes in the first 20 min of incubation.

**Figure 8:**
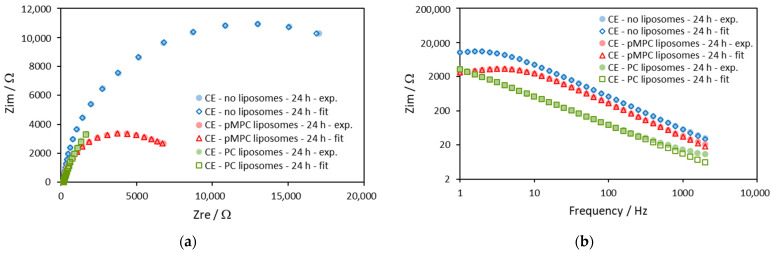
Impedance diagrams on carbon electrodes at the 24th hour of equimolar solution of 0.5 mM ferricyanide (Fe(CN)_6_^3−^) and 0.5 mM ferrocyanide (Fe(CN)_6_^4−^) in the absence (blue symbols) and in the presence of pMPC liposomes (red symbols) and PC liposomes (green symbols). Solid symbols—experimental data; open symbols—fitting data. Screen-printed carbon electrodes: DRP-110; surface area, 0.1257 cm^2^. Supporting electrolyte: PBS. Frequency range: 1–2000 Hz. DC potential: 95 mV. (**a**) Nyquist plot—Z_im_ vs. Z_re_; (**b**) Bode plot—Z_im_ vs. Frequency.

The impedances on carbon electrodes obtained after 24 h of incubation are shown in Figure 8.

In Figure 8, we can see that the difference in the impedance diagrams is quite noticeable after 24 h of incubation for all three cases.

Comparing the semicircles in Figure 8a, it is noted that the semicircle obtained with pMPC liposomes in solution has a much smaller diameter, which means a much smaller charge transfer resistance *R*_ct_ compared to the results in PBS and in PC liposomes solution. The data of the electrode-incubated PC liposomes (green symbols) look like a section of a semicircle with a diameter closer to the electrode in PBS. In the higher frequencies of the Bode plot (Figure 8b), the curve of the electrode with the pMPC liposomes is beneath the one without liposomes, meaning higher apparent capacitance with pMPC liposomes. At the same time, the curve of the Bode plot of the electrode with the PC liposomes is even lower, suggesting even higher capacitance in this case.

The parameters of the impedances obtained by the approximation with the model are given in Table 4.

It is observed that after 24 h of incubation, there is a substantial increase of the *Q* parameter for all electrodes in the three solutions—without liposomes, with pMPC liposomes, and with PC liposomes. Since this change is observed in the solution without liposomes, the increase of *Q* in PBS is primarily due to some interaction with the content of the supporting PBS electrolyte. The increase of *Q* is due to the increase of the electric double-layer capacitance and, hence, should be related to some adsorption of the ions of the PBS. Eventually, this adsorption also prevents, to some extent, the close contact of the electroactive ions (Fe(CN)_6_^3−^ and Fe(CN)_6_^4−^) to the electrode surface, which results in the slower electron transfer observed in the voltammogram and the bigger *R*_ct_ in solution without liposomes after 24 h of incubation. It is noted that the *Q* parameter is even bigger, and *R*_ct_ is smaller with pMPC liposomes, suggesting that there is some kind of interaction between the carbon surface and the pMPC liposomes. The results can be explained by physical adsorption of the pMPC liposomes on the carbon electrode surface without the formation of chemical bonds or additional fusion and spreading of pMPC liposomes and the formation of lipid film. As previously stated, the formation of a lipid film on the surface or a film of any kind of chemically adsorbed species would result in a decrease of *Q* and an increase of *R*_ct_, changes that are exactly opposite to the observed ones for the electrode incubated with pMPC liposomes. As in the case of gold electrodes, the physical adsorption of the highly hydrated pMPC liposomes leads to a higher concentration of the electroactive ions (Fe(CN)_6_^3−^ and Fe(CN)_6_^4−^) close to the electrode surface that results in the smaller *R*_ct_ compared to the electrode incubated in PBS. The physical adsorption of the pMPC liposomes with its highly hydrated surface also results in better hydration of the carbon surface, which could also explain the higher *Q* parameter and the more pronounced electron transfer characteristics as the better hydration results in bigger capacitance of the electrode electric double layer and closer contact of the hydrophilic electroactive species.

In contrast to the pMPC liposomes, the PC liposomes interact strongly with the carbon electrode surface and in a manner that is different from the PC liposomes’ interaction with the gold electrodes. The *Q* parameter is an order of magnitude higher compared to both carbon electrodes incubated in solutions without liposomes and with pMPC liposomes. This is exactly opposite to the changes observed on gold electrodes. At the same time, the *R*_ct_ values are almost the same for carbon electrodes incubated in solution without liposomes and in solution with PC liposomes. The most plausible explanation is that the PC liposomes rupture on the surface of the carbon electrode but do not form a uniform lipid layer on its surface. The individual lipid molecules are probably in close contact with the carbon electrode surface but in a disordered manner that does not prevent the contact of the hydrophilic electroactive species Fe(CN)_6_^3−^ and Fe(CN)_6_^4−^ with the electrode surface. A close contact with the dipole polar heads of the PC molecules with the electrode surface could explain the observed very high *Q* parameter and the very high apparent capacitance. The observed decrease in the α parameter of the CPE element in the model also suggests a disordered electrode surface that could be due to individually adsorbed PC molecules or to PC liposomes randomly ruptured on the electrode surface without forming a uniform lipid layer.

**Figure 9:**
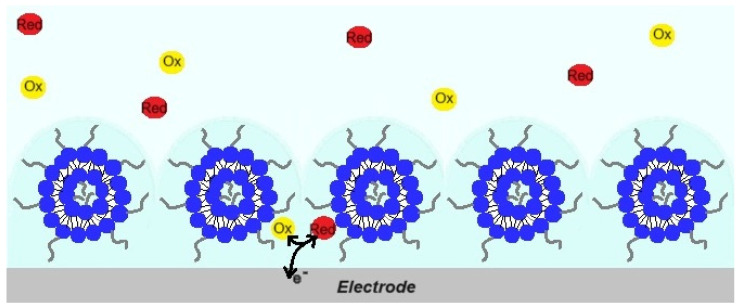
Schematic illustration of pMPC liposomes contacting the electrode surface. The pMPC liposomes do not fuse and spread on the surface, allowing the red-ox electroactive species Red (Fe(CN)_6_^4−^) and Ox (Fe(CN)_6_^3−^) to reach the surface and exchange electrons with the electrode.

Summarizing the results reported for the interaction of the pMPC liposomes and of PC liposomes with the surfaces of gold and carbon screen-printed electrodes, we can propose the following schematic presentation of the interaction of the pMPC liposomes (Figure 9).

## 3. Materials and Methods

### 3.1. Preparation of Liposomes

pMPC liposomes and PC liposomes were prepared as unilamellar spherical vehicles in the sub-micron size range. The preparation process involved downsizing spontaneously formed multilamellar large vesicles (MLVs) by membrane extrusion under high pressure. The liposomes were diluted in phosphate buffer saline (PBS) at a physiological pH.

Briefly, pMPC liposomes were prepared from phosphatidylcholine (PC) lipids and lipid–polymer conjugate (LPC) dissolved in ethanol at a molar ratio of 99.3:0.7. The LPC was prepared by covalent substitution of poly[2-(methacryloyloxy)ethyl phosphorylcholine] to distearoylphosphatidylethanolamine (PSPE, Lipoid Ludwigshafen, Ludwigshafen, Germany) via atom transfer radical polymerization as previously described [9]. The PC/LPC solution is hydrated with PBS above the membrane transition temperature to form MLVs. The obtained water–ethanol mixture is filtered through a 0.2 µm filter by extrusion to downsize the MLVs to unilamellar sub-micron spheres. PC liposomes were prepared using the same protocol from phosphatidylcholine lipids only.

Tangential flow filtration (TFF) removes the ethanol, and the liposomal solution is diluted to the required concentration.

### 3.2. Characterization of the Liposomes—Sizing by Dynamic Light Scattering (DLS) and Zeta Potential Measurements

Samples are diluted to 1 mM total lipid with PBS for both measurements and characterized using ZetaSizer Nano ZS (Malvern Instruments, Ltd., Worcestershire, UK). For the determination of the hydrodynamic diameters, the liposome suspensions were transferred to a disposable polystyrene cuvette. The sample is allowed to equilibrate to 25 °C for 90 s, and three measurements are performed continuously. The autocorrelation function is analyzed by the cumulant method to produce the mean particle hydrodynamic diameter (z-average) and the polydispersity index (PdI). Zeta potential measurements were performed in a disposable folded capillary cell with integrated gold-plated electrodes. The cuvette was placed in the instrument and allowed to equilibrate at 25 °C for two minutes. Three consecutive measurements are performed for each sample. The duration of each measurement was automatically determined by the instrument according to the signal quality.

### 3.3. Electrodes

To investigate the interaction between pMPC liposomes and electrode solid surfaces two different types of electrodes were used. Both were commercially available screen-printed electrodes produced by Metrohm DropSens (Oviedo, Asturias, Spain) and ready for use. In screen-printed electrodes, the working electrode, the reference electrode, and the counter electrode are all “printed” on a ceramic support.

The first type of electrode was screen-printed carbon electrodes (ref. DRP-110). This type of working electrode is made of carbon. The diameter of the electrode is 4 mm, so the working area is 0.1257 cm^2^. The auxiliary counter electrode is also made of carbon. The reference electrode is silver.

The second type of electrode was screen-printed gold electrodes (ref. DRP-220AT). This type of working electrode is made of gold. The diameter of the electrode is 4 mm, so the working area is 0.1257 cm^2^. The auxiliary counter electrode is also made of gold. The reference electrode is silver.

### 3.4. Electrochemical Measurements

All types of experiments were performed with parallel measurements with two types of solutions, one without liposomes and one with liposomes. The supporting electrolyte solution was PBS (Dulbecco’s phosphate buffered saline without calcium and magnesium, pH 7.4).

For the experiments with liposomes, a stock solution of pMPC liposomes 10 mm in PBS (Dulbecco’s phosphate buffered saline without calcium and magnesium, pH 7.4) was used. A small aliquot of this stock solution was taken and dissolved to the supporting electrolyte to reach a final concentration of 80 μL/mL (resulting in 0.8 mm liposomes in PBS).

Potassium ferricyanide K_3_[Fe(CN)_6_] and potassium ferrocyanide K_4_[Fe(CN)_6_] were used as an electroactive couple for probing the electron transfer through the electrode surface and the effect of liposomes on it. Small aliquots of solutions of K_3_[Fe(CN)_6_] and K_4_[Fe(CN)_6_] were added to the supporting PBS electrolyte to give equimolar concentrations of the two. The final concentrations were in the range of 0.125 mm to 1 mm and are mentioned for each experiment in the Section 2.

The impedance data were fitted with the electrical model shown in Figure 3.

The electrical model comprises the resistance of the electrolyte solution *R*_sol_ in series with the working electrode impedance that combines a constant phase-angle element (CPE), a charge transfer resistance *R*_ct_ and a Warburg impedance *Z_w_*.

The impedance of the CPE is as follows [27]:

(1) $$Z_{CPE}=\frac{1}{Q{\left(j\omega \right)}^{\alpha }}$$

Here, *j* = (−1)^1/2^ is the imaginary unit, ω = 2π*f* is the angular frequency, and *f* is the frequency. The CPE coefficient *Q* and the CPE exponent *α* are the characteristics of the CPE. The CPE exponent *α* can take values from 0 to 1. For *α* = 0, the CPE is a pure resistor with resistance *R* = 1/*Q*. If *α* = 1, the CPE becomes an ideal capacitor with capacitance *C* = *Q*.

It should be mentioned that at any frequency, the *Z*_CPE_ can be presented as a combination of apparent parallel capacitance and resistance that are related to *Q* and α [27]:

(2) $$C_{CPE}=Q\sin\left(\frac{\alpha \pi }{2}\right)\omega^{\alpha -1}$$

(3) $$R_{CPE}=\frac{\omega^{-\alpha }}{Q\cos\left(\frac{\alpha \pi }{2}\right)}$$

The CPE behavior is generally thought to arise from the presence of some inhomogeneities in the electrode–material system and could be associated with either surface or normal time constant (RC) distributions [36]. These inhomogeneities are usually related to the roughness of the solid electrodes, but it was also demonstrated that the CPE behavior on solid electrodes could be due to the atomic scale inhomogeneities [37,38]. On the Nyquist plot, the CPE behavior results in the displacement of the origin of the semicircle below the x-axis [39].

When the impedance is measured at the DC potential of the working electrode equal to the equilibrium potential of the electroactive couple species, *E*_0_, the charge transfer resistance is determined as follows [12]:

(4) $$R_{ct}=\frac{RT}{nFi_{0}}$$

where *i*_0_ is the exchange current at *E*^0^, *R* and *F* are the gas constant and the Faraday constant, *T* is the temperature, and n is the stoichiometric number of electrons involved in an electrode reaction.

The Warburg impedance is given as follows [12]:

(5) $$Z_{W}=\sigma \omega^{-1/2}-j\sigma \omega^{-1/2}$$

where *ω* is the angular frequency of the applied alternate potential (*ω* = 2π*f*), *j* is the imaginary unit, and the parameter *σ* is equal to [12]:

(6) $$\sigma =\frac{RT}{F^{2}A\sqrt{2}}\left(\frac{1}{D_{O}^{1/2}C_{O}^{*}}+\frac{1}{D_{R}^{1/2}C_{R}^{*}}\right)$$

Here, *R* and *F* are the gas constant and the Faraday constant, *T* is the temperature, *A* is the surface area of the electrode, *D*_O_ and *D*_R_ are the diffusion coefficients, and *C*_O_^*^ and *C*_R_^*^ are the bulk concentrations of the oxidized and reduced form of the species.

The data from our experiments were fitted with commercially available software ZSympWin 3.60, Echem Software 3.60, USA. In this software, the impedance of the Warburg element is presented with an equation that has a different form compared to Equation (5), where instead of the parameter *σ*, a parameter *W* is used:

(7) $$Z_{W}=W^{-1}{\left(j\omega \right)}^{-1/2}$$

The relationship between the two parameters *σ* and W is as follows:

(8) $$W=\frac{\cos\left(\pi /4\right)}{\sigma }$$

## 4. Conclusions

The reported CV and EIS experiments suggest assuredly that pMPC liposomes do not fuse and spread and do not form any lipid film on the surfaces of carbon and gold electrodes.

In case of formation of a lipid film on the electrode surfaces through fusion and spreading a decrease of the peak currents and displacement of the peak potentials to greater overpotentials would be observed in the CV experiments. A significant decrease of the apparent capacitance and increase of the charge transfer resistance would be observed in the EIS experiments which was shown for PC liposomes interacting with the gold electrode surface.

None of these changes were observed for pMPC liposomes. On the contrary, all parameters in both CV and EIS do change in the opposite direction. During the first 20 min of incubation, changes are not observed or are considered insignificant. After 24 h of incubation, the charge transfer characteristics are better with pMPC liposomes in the solution, which is demonstrated in both CV and EIS experiments. In CV, the peak currents are bigger with both carbon and gold electrodes. The peak potentials are the same as those of gold electrodes and even closer to the equilibrium potential with carbon electrodes. In EIS, the apparent capacitance, represented by the parameters *Q* and *α*, is bigger with pMPC liposomes on both electrodes, with a more significant difference with the carbon electrodes. The charge transfer resistance, *R*_ct_, is smaller with pMPC liposomes on both electrodes, with a more significant difference again with the carbon electrodes.

The observed results could be explained by the physical adsorption without additional fusion and spreading of the pMPC liposomes on the surfaces of the electrodes. The increased capacitance and decreased charge transfer resistance could be explained by the highly hydrated surface layer around the pMPC liposomes that brings more charged electroactive species close to the electrodes’ surfaces. According to the results, the adsorption of pMPC liposomes is more prominent on the carbon electrode surface.

The results also do not support an occurrence of chemical adsorption of liposomes without additional spreading and lipid film formation. The chemical bonds would decrease the sites on the electrode surface available for the electron charge transfer, and the charge transfer resistance R_ct_ would be greater with pMPC liposomes, which is not the case with our experiments.

## 5. Patents

All patents related to the technology presented in this publication are owned or licensed by Liposphere Ltd., HaMerkaz, Israel.

## Figures and Tables

**Table 1:** Parameters of the impedance model calculated by the fitting procedure for gold electrode impedances measured in the absence of liposomes and in the presence of pMPC liposomes at the 20th minute. Conditions of measurement as mentioned in the Figure 2 caption.

Gold Electrode—20 min	*R*_sol_/Ω	*Q*/Ω^−1^s^α^	*α*	*R*_ct_/Ω	*W*/Ω^−1^s^0.5^
no liposomes	30.05	4.118 × 10^−6^	0.9356	598.7	2.659 × 10^−4^
with pMPC liposomes	29.89	3.904 × 10^−6^	0.9532	512.7	2.924 × 10^−5^

**Table 2:** Parameters of the impedance model calculated by the fitting procedure for gold electrode impedances measured in the absence of liposomes and in the presence of pMPC liposomes and PC liposomes at the 24th hour. Conditions of measurement as mentioned in the Figure 4 caption.

Gold Electrode	*R*_sol_/Ω	*Q*/Ω^−1^s^α^	*α*	*R*_ct_/Ω	*W*/Ω^−1^s^0.5^
no liposomes—0 min	30.51	3.570 × 10^−6^	0.9539	117	28.3 × 10^−5^
no liposomes—24 h	32.66	3.237 × 10^−6^	0.9393	15,820	10.6 × 10^−5^
with pMPC liposomes—24 h	35.44	3.207 × 10^−6^	0.9705	12,190	11.4 × 10^−5^
with PC liposomes—24 h	32.64	1.141 × 10^−6^	0.9272	92,740	0.53 × 10^−5^

**Table 3:** Parameters of the impedance model calculated by the fitting procedure for carbon electrode impedances measured in the absence of liposomes and in the presence of pMPC liposomes at the 20th minute. Conditions of measurement as mentioned in the Figure 7 caption.

Carbon Electrode—20 min	*R*_sol/_Ω	*Q*/Ω^−1^s^α^	*α*	*R*_ct/_Ω	*W*/Ω^−1^s^0.5^
no liposomes	157.7	1.263 × 10^−6^	0.9705	7216	2.397 × 10^−4^
with pMPC liposomes	171.6	1.370 × 10^−6^	0.9653	6687	2.518 × 10^−4^

**Table 4:** Parameters of the impedance model calculated by the fitting procedure for carbon electrode impedances measured in the absence of liposomes and in the presence of pMPC liposomes and of PC liposomes at the 24th hour. Conditions of measurement as mentioned in the Figure 8 caption (0.5 mM ferri/ferrocyanide).

Carbon Electrode	*R*_sol_/Ω	*Q*/Ω^−1^s^α^	*α*	*R*_ct_/Ω	*W*/Ω^−1^s^0.5^
no liposomes—0 min	189.2	1.170 × 10^−6^	0.9701	10,043	2.03 × 10^−4^
no liposomes—24 h	198.3	3.782 × 10^−6^	0.9658	20,920	1.14 × 10^−4^
with pMPC liposomes—24 h	203.4	5.569 × 10^−6^	0.9767	6302	2.35 × 10^−4^
with PC liposomes—24 h	184.8	54.77 × 10^−6^	0.8449	19,540	8.42 × 10^−4^

## Data Availability

The data are available upon request to the authors.

## Supplementary Materials

The following supporting information can be downloaded at: https://www.mdpi.com/article/10.3390/molecules29153511/s1, Figure S1: Fluorescence emission spectra of NBD-PE at different stages of the Fluorescence quenching lamellarity assay (excitation at 465 nm) for the pMPC liposomes incorporating NBD-PE; Figure S2: Normalized emission intensity curve of the emission peak of NBD-PE before and after addition of dithionite and after liposome disassembly by Triton X-100 and heating.
