# Supplementary material for: Electrochemical Investigation of the Stability of Poly-Phosphocholinated Liposomes
Source: Molecules. 2024 Jul 26;29(15):3511. doi: 10.3390/molecules29153511 (PMC11313893; doi:10.3390/molecules29153511)
Supplement: Supplementary file 1 [file molecules-29-03511-s001.zip › molecules-3083060-supplementary.pdf]

Supplementary Material

Lamellarity assay by fluorescence quenching:

The lamellarity of the liposomes was investigated applying a fluorescence quenching assay [Ref. 31]. Briefly, a liposomal formulation incorporating the fluorescent lipid NBD-PE at 0.5 mol% of total lipids was prepared using the same protocol as the test samples. Fluorescence emission was recorded on a Synergy H1 (S/N 1902141A) plate reader. Samples were diluted with PBS to reach signal levels that are within the detection range of the instrument. Measurements were performed on 200 μL sample volume in a 96-well plate with excitation at 465 nm. The emission of NBD-PE was recorded between 550 and 670 nm. Then, 2 μL of a 500 mM stock solution of the quencher dithionite were added to the sample to achieve 5 mM final concentration followed by mixing using a pipette. The emission was recorded until no more quenching of the solvent exposed fluorophores was observed. Finaly, 20 μL of a 10% w/w Triton X-100 were added to disassemble the liposomes followed by heating to 65°C, above the phase transition temperature of the lipid bilayer. In the liquid phase the liposomal membranes are completely disassembled. After this treatment, the fluorescence was quenched completely indicating quencher accessibility to fluorophores in the inner volume of the liposome.

Figure S1 and Figure S2 present the results of the lamellarity test of pMPC liposomes labelled with NBD-PE. Addition of dithionite to a final concentration of 5mM, quenched the fluorescence emission of the solvent exposed lipids by 43%, indicating a highly unilamellar formulation. Further addition of Triton X-100 to a final concentration 1%, disassembled the liposomal structure and allowed complete quenching of all (Figure S2).

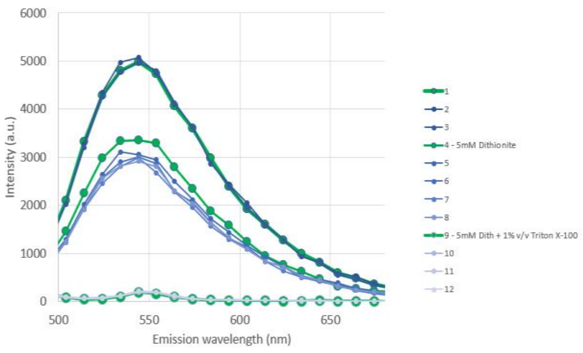

Figure S1: Fluorescence emission spectra of NBD-PE at different stages of the Fluorescence quenching lamellarity assay (excitation at 465nm) for the pMPC liposomes incorporating NBD-PE.

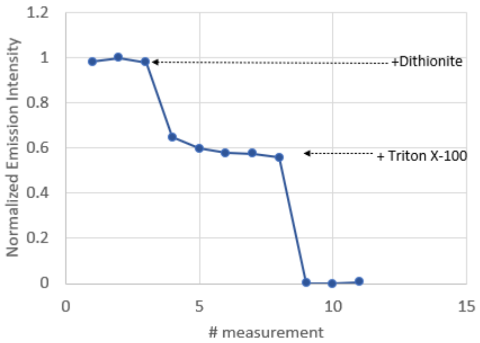

Figure S2: Normalized emission intensity curve of the emission peak of NBD-PE before and after addition of dithionite and after liposome disassembly by Triton X-100 and heating.
